# Information elaboration and epistemic effects of diversity

**DOI:** 10.1007/s11229-019-02108-w

**Published:** 2019-02-15

**Authors:** Daniel Steel, Sina Fazelpour, Bianca Crewe, Kinley Gillette

**Affiliations:** 1 W. Maurice Young Centre for Applied Ethics, University of British Columbia, 6356 Agricultural Road, Vancouver, BC V62 1Z2 Canada; 2 Department of Philosophy, University of British Columbia, 1866 Main Mall, Vancouver, BC V6T 1Z1 Canada

**Keywords:** Diversity, Science, Social epistemology, Modelling, Epistemic injustice

## Abstract

We suggest that philosophical accounts of epistemic effects of diversity have given insufficient attention to the relationship between demographic diversity and information elaboration (IE), the process whereby knowledge dispersed in a group is elicited and examined. We propose an analysis of IE that clarifies hypotheses proposed in the empirical literature and their relationship to philosophical accounts of diversity effects. Philosophical accounts have largely overlooked the possibility that demographic diversity may improve group performance by enhancing IE, and sometimes fail to explore the relationship between diversity and IE altogether. We claim these omissions are significant from both a practical and theoretical perspective. Moreover, we explain how the overlooked explanations suggest that epistemic benefits of diversity can depend on epistemically unjust social dynamics.

## Introduction

The effect of diversity on science is an active topic of research among philosophers (Harding 2015; Intemann 2010; Kitcher 1990; Longino 1990, 2002; Muldoon 2013; Solomon 2001; Steel et al. 2018; Zollman 2010). Some recent contributors to this subject argue that philosophical models of diversity in science should be more closely linked to the target systems they purport to describe (Martini and Pinto 2016; Grim et al. 2019). An important step in this direction is to examine empirical research on how diversity affects group performance. However, doing this is hampered by the sparsity of empirical research on the effects of diversity in science, especially along demographic lines such as gender or ethnicity (Hall et al. 2018, p. 541). But there is a large and rich empirical literature examining the effects of diversity on group performance in areas outside of academic science, such as business and management. While it would be unwise to assume that such findings transfer without modification to science, it would also be highly inefficient to disregard this work altogether. That is especially the case for research on explanations of how diversity impacts the performance of groups or teams.^1^ An explanation that has empirical support in one social context is reasonably taken as a possibility to be tested in others, especially when the underlying psychological or social mechanisms appear fairly general. Consequently, the present paper examines empirical research on diversity both within and without science, and explores its implications for accounts of diversity in philosophy of science.

Explanations of the effects of diversity are often framed by the trope of a “double-edged sword” (Bear and Wooley 2011; Hall et al. 2018, pp. 535–536; Horwitz and Horwitz 2007; Milliken and Martins 1996; Smith-Doerr et al. 2017). On the positive edge, distinct social backgrounds may be associated with differing perspectives or experiences relevant to a task, and the joint consideration of these may result in superior options being discovered, implemented, or agreed upon. On the negative edge, demographic diversity may generate obstacles to communication and trust, which may impair group performance. Although influential, the double-edged sword is not without its critics. A key component of the double-edged sword is that if demographic diversity has positive effects on performance, it does so because who you are makes a difference to your task-related knowledge (Page 2017, p. 169). But some critics (e.g., Phillips 2017) argue for an additional route: demographic diversity can improve performance by enhancing information elaboration (IE), the process through which information and perspectives distributed among the group are elicited and assessed (van Homan et al. 2007; van Kooij-de Bode et al. 2008; Meyer et al. 2011). For convenience, we use the label *diversity can benefit information elaboration* (DBIE) to refer to such explanations. DBIE explanations differ from the double-edged sword in claiming that demographic diversity can improve group performance even when it is not linked to the possession of distinctive information or perspectives related to the task, and that it can have positive, as well as negative, impacts on IE.

We suggest that empirical diversity research has important implications for philosophical discussions of diversity in science. A number of philosophers have developed formal models to study epistemic effects of cognitive diversity, but these models generally do not consider the effects of diversity on IE, which restricts their ability to study potential negative effects of diversity suggested by the double-edged sword and positive effects proposed by DBIE explanations. We also consider Longino’s account of diversity and scientific objectivity. While Longino’s proposal is sensitive to potential negative effects of diversity upon IE, we argue that it does not consider DBIE explanations. Several significant implications follow from these observations. One is to identify explanatory and practical limitations to the approaches we consider, and to suggest lines of research that may address them. Moreover, DBIE explanations imply that some epistemic benefits of diversity may depend on phenomena related to social injustices, such as stereotyping or discounting the testimony of out-group individuals. We argue that this entails a more complex relationship between epistemic and justice-based rationales for diversity in science.

The organization of this paper is as follows. Section 2 describes empirical literature regarding diversity impacts on group performance, using the double-edged sword as an organizing device. Section 2.1 focuses on the double-edged sword, while Sect. 2.2 examines DBIE explanations. The latter requires developing a more precise account of IE. We propose that IE consists of communication, integration, and iteration in a context of dispersed cognitive resources. Section 3 examines formal models of diversity in science and Longino’s (1990, 2002) account of diversity and objectivity from the perspective of IE. The final section offers concluding remarks.

## Explanations of diversity impacts

As Hall and colleagues write, “There is a paucity of literature on diversity in science teams, particularly when cultural, national, and racial/ethnic diversity are concerned” (Hall et al. 2018, p. 541). Consequently, we examine diversity research both within and without science, although we consider commonalities and differences between these domains. Section 2.1 uses the double-edged sword as a backdrop for discussing some central findings and explanations regarding the effect of diversity on group performance. Section 2.2 describes two explanations that lie off the well trodden path of the double-edged sword.

### The double-edged sword

According to Milliken and Martins, “Diversity… appears to be a double-edged sword, increasing the opportunity for creativity as well as the likelihood that group members will be dissatisfied and fail to identify with the group” (Milliken and Martins 1996, p. 403). Statements of the double-edged sword frequently add several further features. Consider this passage from Hong and Page, who present a formal model intended to clarify conditions under which functional diversity enhances group performance:

The claim that perspectives and heuristics may be influenced by race, geography, gender, or age has much to recommend it, as does the claim that perspectives and tools are shaped by experiences, training, and preferences. However, even when applying our result to those cases when identity diversity has been shown to correlate with functional diversity, we need to be acutely aware that identity-diverse groups often have more conflict, more problems with communication, and less mutual respect and trust among members. (Hong and Page 2004, pp. 16385–16386)

**Fig. 1:**
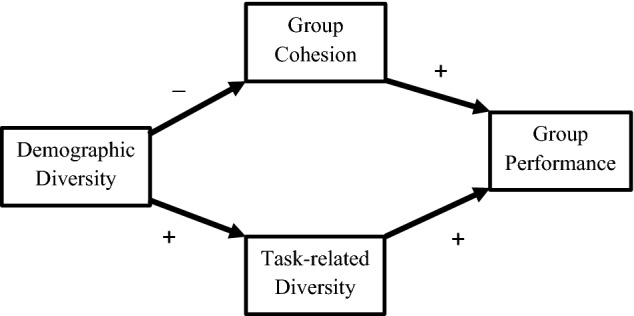
The double-edged sword represented by a directed acyclic graph

This statement is typical of formulations of the double-edged sword insofar as it: (a) distinguishes diversity of demographics or social identity from diversity of task-relevant cognitive characteristics, (b) suggests that positive effects of demographic diversity are mediated by task-related diversity, and (c) attributes negative effects on group cohesion to demographic diversity (cf. Hülsheger et al. 2009; Joshi and Roh 2009, p. 600; Lungeanu and Contractor 2015; Reagans and Zuckerman 2001, p. 506; Smith-Doerr et al. 2017; Stahl et al. 2010; van Dijk et al. 2012, p. 39). The causal structure of such formulations of the double-edged sword is represented by the graph in Fig. 1.

Demographic diversity refers to diversity in demographic categories, such as gender, nationality, ethnicity, race, or religious affiliation, whereas task-related diversity refers to diversity in characteristics presumed to be more directly relevant to the task in question, such as education, experience, or expertise (Horwitz and Horwitz 2007, p. 990). Similar distinctions are drawn in several ways in the literature, including identity versus cognitive or shallow versus deep (see Hong and Page 2004; Horwitz and Horwitz 2007; Joshi and Roh 2009; Lungeanu and Contractor 2015; Milliken and Martins 1996; Page 2017; Phillips 2017; Smith-Doerr et al. 2017; Stahl et al. 2010; van Dijk et al. 2012). We adopt the demographic versus task-related distinction here, as this appears to be the most commonly used. Group cohesion refers to the cluster of communication, mutual respect, trust, and identification with the group that, according to the double-edged sword, enhances group performance but is inhibited by demographic diversity (cf. Reagans and Zuckerman 2001, p. 512). Finally, as the label suggests group performance refers to how well the group has achieved the assigned task. As noted below, measuring group performance raises methodological complexities, as different types of measurement can produce distinct types of bias.

The graph in Fig. 1 is helpful for appreciating empirical predictions of the double-edged sword. Since demographic diversity does not determine task-related diversity, the double-edged sword entails that group performance is more positively (or less negatively) associated with the latter than the former. That is, according to the double-edged sword, all positive effects of demographic diversity on group performance are mediated by task-related diversity. Thus, the positive association between demographic diversity and group performance is more attenuated than between task-related diversity and group performance. Similarly, task-related diversity is less closely linked than demographic diversity to the negative impacts running through the pathway mediated by cohesion. Note that the double-edged sword does not necessarily predict that the association between group performance and task-related diversity is positive, although it is consistent with this result. Whether this association is positive or negative depends on the relative strengths of the two counteracting paths.

Consider a study that illustrates the predictions of the double-edged sword. Lungeanu and Contractor (2015) study the effect of diversity on innovation, measured by publication counts, among research teams in the newly emerging field of oncofertility.^2^ They find that innovation is positively associated with knowledge diversity, not associated with gender diversity, and negatively associated with international diversity (Lungeanu and Contractor 2015, pp. 556–557). Moreover, in keeping with the negative edge of the double-edged sword, Lungeanu and Contractor suggest that greater international collaboration suppresses innovation by inhibiting group coordination and communication (Lungeanu and Contractor 2015, p. 551).

However, some studies find positive effects for demographic diversity. For example, Campbell et al. (2013) find a positive association between gender diversity among author teams of scientific articles and number of citations (cf. Freeman and Huang 2014). The tendency for studies to generate differing results about group performance is often attributed to effect modifiers that vary across contexts.^3^ Many such effect modifiers have been proposed and studied (Eagly 2016; Guillaume et al. 2017; van Dijk et al. 2012). Some are suggested by agent-based models that identify conditions under which cognitive diversity improves group performance, such as task complexity and competence of problem solvers for the task (Hong and Page 2004; Page 2007, 2017). In addition, some propose that effects of increased diversity are more positive when the group distribution is more balanced and more negative when it is more homogeneous (Bear and Wooley 2011; Joshi and Roh 2009; Joshi et al. 2011; Reagans and Zuckerman 2001; Smith-Doerr et al. 2017). Other effect modifiers thought to enhance positive effects of demographic diversity include positive attitudes toward diversity (van Homan et al. 2007; Mitchell and Boyle 2015; van Knippenberg et al. 2013), the presence of egalitarian rather than hierarchical social relationships (Smith-Doerr et al. 2017; Schneid et al. 2015), and the absence of fault lines, that is, multiple aligning differences, as would be present in a group composed of white male doctors and black female nurses (Meyer and Schermuly 2012).

Despite variability of individual studies, literature reviews that examine demographic and task-related diversity usually find a more positive effect for the latter, as the double-edged sword predicts. Literature reviews typically find a small positive association between group performance and task-related diversity (Bell et al. 2011; Eagly 2016; Hülsheger et al. 2009; Horwitz and Horwitz 2007; Mello and Rentsch 2015).^4^ In comparison, effects of demographic diversity on group performance tend to be more mixed, and negligible or even slightly negative when aggregated in meta-analyses (Bear and Wooley 2011; Bell et al. 2011; Eagly 2016; Horwitz and Horwitz 2007; Hülsheger et al. 2009; Joshi and Roh 2009).^5^ However, some have challenged the conventional wisdom that performance is more positively associated with task-related diversity than with demographic diversity.^6^ A meta-analysis by Van Dijk et al. (2012) finds this pattern for subjective measures of performance (e.g., assessments of team performance by supervisors) but not for objective measures (e.g., number of papers published, patents, earnings, etc.). But for highly complex tasks and tasks requiring innovation, a statistically significant positive association was found between objective measures of performance and task-related but not demographic diversity (van Dijk et al. 2012, pp. 44–46).

The vast majority of diversity research covered in the systematic reviews and meta-analyses cited above comes from fields outside of science, which raises questions about its relevance to scientific teams. An important commonality is that the double-edged sword also frequently arises in discussions of diversity in team science (Freeman and Huang 2015, p. S301; Hall et al. 2018, p. 536; Lee et al. 2015; Lungeanu and Contractor 2015; Stvilia et al. 2010). Indeed, the double-edged sword is evident in some feminist philosophy of science, which emphasizes the potential for increased gender diversity to elicit both sexist discrimination and helpful new perspectives (Fehr 2011; Intemann 2011). However, differences also exist. One has to do with distinct biases resulting from different ways of measuring performance. Subjective assessments of performance by group members or supervisors are common in diversity research in business and organization fields, which may create a bias against demographic diversity if people harbor negative stereotypes regarding outgroups (van Dijk et al. 2012). In contrast, in team science research, performance is usually measured using bibliometrics, such as number of citations (Hall et al. 2018, p. 540), which can generate opposite biases. For instance, diverse teams might garner more citations because they are more likely to include prominent researchers or have a broader professional network (Freeman and Huang 2015, p. S302; cf. Hall et al. 2018, p. 542).

### Off the beaten path: diversity can benefit information elaboration (DBIE)

This section examines explanations that challenge a central feature of the double-edged sword, expressed vividly by Page as follows:

For cognitive diversity to produce a bonus, it must be germane to the task. That same logic applies to identity diversity. For women, by virtue of being women, to create immediate diversity bonuses, women’s repertoires—their knowledge, information, models, heuristics, and representations—would have to produce more accurate predictions, more creative ideas, better solutions to problems, or more comprehensive evaluations of projects. (Page 2017, p. 169)

In other words, any benefits flowing from demographic diversity to group performance are mediated by cognitive diversity specific to the content of the task. This section considers the idea that demographic diversity can benefit group performance through another route, namely, by improving information elaboration (IE).

It is well-known that the mere presence of relevantly diverse information or perspectives is not sufficient to ensure improved performance on a group task.^7^ It is also necessary that these diverse cognitive resources be elicited, assessed, and brought to bear on the task at hand. The term “information elaboration” is used to refer to this process (van Homan et al. 2007; van Kooij-de Bode et al. 2008; Meyer et al. 2011). Homan and colleagues define IE as, “the exchange of information and perspectives, individual-level processing of the information and perspectives, feeding back the results of this individual-level processing into the group, and discussion and integration of their implications” (van Homan et al. 2007, p. 1189). As IE plays an important role in the ensuing discussion, it will helpful to examine this concept more closely.

Let the term *cognitive resources* refer to beliefs, perspectives, heuristics, methods or any other task-relevant cognitions or abilities. A cognitive resource is *at the disposal* of an individual if that individual is able to combine it with other cognitive resources to derive results. Examples include drawing logical inferences by conjoining one’s own beliefs with newly communicated information, and applying one’s methods or heuristics to a newly posed problem. Finally, *dispersed cognitive resources* are present when some cognitive resources are at the disposal of some but not other group members. In this context, IE can be characterized as consisting of the following three steps: (1) *communication* of dispersed cognitive resources, (2) *integration* in which individual group members generate results by conjoining communications with the cognitive resources at their disposal, and (3) *iteration* of the first two steps given new resulting dispersed cognitive resources, such as inferences drawn by individuals from newly shared information.

To illustrate, consider a group working to improve a website reader software package. Suppose this group includes, among others, a person with visual impairment, A, and an expert on technical aspects of website reader software, B. Suppose that A has encountered a difficulty when using the website reader of which others in the group are unaware. Then the following would be an example of IE. First, A communicates the difficulty. Then other group members consider what consequences follow from it given their own knowledge; for instance, B considers how the software could be redesigned to avoid the difficulty. Next, group members communicate these inferences, and the group then discusses the suggested ideas, again with individuals considering and communicating implications given their own information and perspectives.

Note that IE does not necessarily involve consensus or collective action. In the manner of philosophical debates, IE might proceed indefinitely without agreement, and IE can occur among individuals pursuing their interests in a marketplace. Nevertheless, IE is often important for groups in which collective action is necessary for successful completion of a task. Thus, the group in the website reader example must eventually agree on a plan for developing an improved version of the software. This example also suggests several possible obstacles to IE. A group member may not communicate relevant information or perspectives. For instance, perhaps A does not feel comfortable discussing a difficulty with the website reader that may offend a prominent group member who takes credit for having designed the software. Or information may be communicated, but others may not explore its consequences because they regard the communicator’s views on the subject as unreliable or unimportant. The double-edged sword hypothesizes that demographic diversity often leads to failures of IE such as these (cf. van Kooij-de Bode et al. 2008).

Unlike the double-edged sword, DBIE explanations propose that demographic diversity can also improve IE. We consider two DBIE explanations, which we label *cognitive diversity expectation* and *in*-*group epistemic conformity*. According to the first, noticeable diversity within the group increases the expectation that cognitive diversity is also present (Holtz and Miller 1985; Phillips 2017, p. 235). These expectations can prime individuals to take note of novel or dissenting views when they are expressed. They can also motivate group members to prepare more thoroughly and to assess information more carefully, as expectation of cognitive diversity suggests that others will not already share one’s information and perspectives (Antonio et al. 2004; Loyd et al. 2013; Sommers 2006). In a homogeneous group, by contrast, the expectation that others possess similar views and information may foster complacency and lead to a failure to adequately process information and perspectives. In terms of IE, these are failures of integration. To illustrate, suppose that group member A asserts P, and that member B has at her disposal—in a document that she alone possesses—information Q that contradicts P. In this case, dissent would occur if B conjoins P with her stock of information, notices the contradiction between P and Q, and then communicates this to others in the group. But if B fails to read the document carefully and consequently fails to notice Q or its logical relation to P, then a failure of integration and a missed opportunity for useful dissent have occurred. In general, cognitive diversity expectation suggests that demographic diversity can enhance IE by reducing the rate of errors and oversights in integration.

The second explanation, in-group epistemic conformity, claims that people are more inclined to agree with, and to desire agreement from, others they perceive as socially similar to themselves (Phillips 2003). This hypothesis is comprised of two interrelated sub-mechanisms: one in which people believe that people like themselves are more likely to hold true beliefs, and a second according to which people have a normative expectation that people like themselves should agree with one another. Thus, in socially homogeneous groups, individuals may put too much trust in the claims of others, be disinclined to express conflicting views, and behave in ways that discourage dissent. For example, some research suggests that people are more likely to be annoyed when dissenting views are expressed by in-group rather than out-group individuals (Antonio et al. 2004; Phillips 2003; Phillips et al. 2004, 2009; Phillips and Loyd 2003). As a result, differences in social identity, even when irrelevant to the task, can lower resistance to considering dissenting views and contrary information (Phillips et al. 2009). Consequently, provided a sufficiently supportive environment, out-group individuals may be more likely to express novel views with greater confidence (Antonio et al. 2004; Swaab et al. 2016). Indeed, the mere presence of out-group individuals can also make in-group individuals more willing to express and consider novel or dissenting views (Phillips et al. 2009; Sommers 2006).

In-group epistemic conformity bears some similarity to the theory of groupthink, according to which extremely cohesive groups suffer epistemically due to suppressing dissent (Tollefsen 2006). It differs, however, in encompassing more subtle and less extreme cases, and consequently need not involve policing of dissent, illusion of group invulnerability, stigmatization of out-groups as the “enemy,” or other characteristics associated with groupthink (cf. Tollefsen 2006, p. 39). In-group epistemic conformity can also result from mechanisms similar to those proposed to explain group polarization, wherein an initial tilt in group option becomes more skewed and extreme due to deliberation (Singer 2018; Sunstein 2002). Individuals may accept claims made by groupmates and discount their own conflicting information, and they may attempt to maintain a positive reputation by preferentially communicating information that reiforces claims of others (Sunstein 2002, pp. 176–177). However, in-group epistemic conformity is distinguished from group polarization in two ways: (a) its focus is on IE, not polarization, and (b) it emphasizes that demographic differences can benefit IE even when not associated with distinctive views or information.^8^

**Fig. 2:**
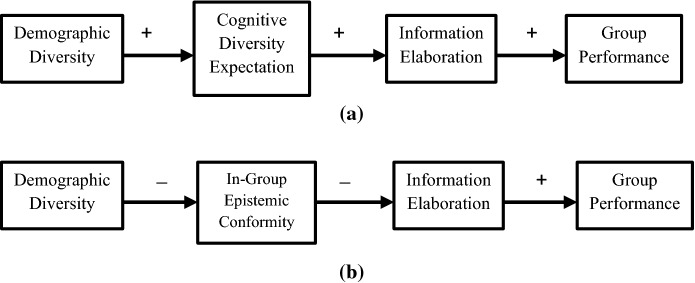
Two DBIE hypotheses. **a** Demographic diversity causes group members to expect cognitive diversity, which changes behaviors in ways that enhance IE. **b** Demographic diversity decreases the tendency for epistemic conformity with other group members, which also improves IE

The double-edged sword and the DBIE explanations in Fig. 2 can be tested head-to-head by experiments in which task-related information is assigned independently of demographics or in which task-related diversity can be accurately measured and statistically controlled for (or some combination of the two). In this setting, DBIE explanations predict that demographic diversity may be positively associated with group performance, while the double-edged sword predicts a negative association. Consider an experiment that illustrates this idea.

Levine et al. (2014) describe an experiment, administered in both South East Asia and North America, in which participants were randomly assigned to trade in an artificial real estate market in an ethnically homogeneous or ethnically diverse condition. All participants were provided with all of the information relevant to assessing the prices of the properties, and individual pricing accuracy was assessed before trading began (Levine et al. 2014, pp. 18525–18526). Participants assigned to the same group briefly sat together in a room prior to trading but could not see one another during the trading process and communicated only via buying and selling properties (Levine et al. 2014, p. 18528). Consider how IE applies to this case. Dispersed cognitive resources are present in the form of differing assessments of the values of properties by distinct research participants. Next, participants communicate these assessments through the quantities of money at which they are willing to buy and sell properties. Other participants observe these transactions, and can integrate them with their own assessments of property values, which can then inform their own decisions. Finally, iteration is present, as prices influenced by previous transactions can impact subsequent ones, and so on.

In both locations, traders in ethnically diverse groups were more likely than those in homogeneous groups to price properties accurately and were less prone to bubbles, a difference that could not be explained by differences in individual trading ability between homogeneous and diverse groups (Levine et al. 2014, p. 18527). Levine and colleagues write, “Ethnic diversity was valuable not necessarily because minority traders contributed unique information or skills, but their mere presence changed the tenor of decision making among all traders” (Levine et al. 2014, p. 18528). Along the lines of the in-group epistemic conformity explanation, Levine and colleagues suggest that this result can be explained by a tendency for people to place more trust in the decisions of socially similar others in comparison to those judged to be socially dissimilar (Levine et al. 2014, p. 18525). That is, individuals in homogeneous groups were more likely to take purchase prices as indicators of the true value of properties and to overlook information at their disposal suggesting that buyers had overpaid. This is a failure of integration.

The explanations examined in this section do not dispute the positive path of the double-edged sword, or that demographic diversity can adversely impact IE in some circumstances. Instead, they claim that demographic diversity can also have positive effects on IE not mediated by task-related diversity. While we think DBIE explanations merit serious consideration, these explanations constitute a relatively small subset of diversity research, making it unclear how robust and replicable results will prove to be. Moreover, we know of no empirical work that examines cognitive diversity expectation or in-group epistemic conformity explanations in connection with scientific teams. However, there are analogies between these explanations and some formal models proposed by philosophers of science, as we explain below.

## Information elaboration in philosophical models of diversity

This section examines philosophical accounts of epistemic effects of diversity. Specifically, we are interested in the extent to which these models are capable of studying the complex relationships between demographic diversity and IE suggested by the double-edged sword and DBIE explanations. In Sect. 3.1, we consider formal models, while Sect. 3.2 considers a widely discussed informal model of diversity in science due to Longino (1990, 2002). We suggest that none of these proposals adequately represent the relationship between demographic diversity and IE, and we consider implications of this limitation.

### Formal models

In this section, we examine some prominent formal models that aim to capture epistemic benefits of diversity. We are interested in whether these models represent the relationship between diversity and IE and what limitations the failure to do so might imply. Consequently, our discussion does not aim to evaluate the adequacy of other aspects of these models.^9^

Our examination of models in this section proceeds in two steps. First, we ask whether the models represent IE. Of those that pass this first hurdle, we can ask whether they can study dependency of IE on demographic diversity, as hypothesized by the double-edged sword or DBIE explanations. In Sect. 2.2, we analyzed IE as involving communication, integration, and iteration in a group with dispersed cognitive resources. Hence, a precondition for IE to even figure in a formal model is that the cognitive resources must vary among agents. Of course, this is compatible with some cognitive similarities. For example, two agents may be identical with one another in their perspectives, but differ in the information they possess. In short, agents need not differ in every way, but there must be some cognitive variation among them. Surprisingly, some formal models designed to study diversity in science lack this feature.

An early formal framework for modeling the epistemic benefits of diversity for science has come to be known as the Marginal contribution/reward (MCR) approach (Kitcher 1990, 1993; Strevens 2003). MCR is motivated by the idea that, under certain conditions, science as a whole would benefit if there is a distribution of labor between different projects, instead of every scientist working on the project with the highest current chance of success (Kitcher 1990). What is more, according to MCR, a group of scientists, who choose their pursuit on the basis of their marginal contribution to a given project and the reward they can expect from that contribution, divide their labor in a way that converges to the optimal division of labor for the scientific collective. However, as noted by Muldoon and Weisberg (2011), the success of MCR models crucially depends on the assumption that, prior to making a choice about which project to pursue, every scientist knows the current distribution of cognitive labor as well as each project’s chance of success, given the current distribution of labor. Accordingly, when it comes to the information they possess, there is nothing that could distinguish one agent from another within the MCR framework. Further, insofar as all scientists are modeled as employing the same rational choice-based strategy for choosing a project, the scientists are also homogeneous with respect to their inferential strategies.

Fortunately, several models designed to study epistemic effects of diversity represent agents as possessing varying cognitive resources. One way is to adopt a heuristic search approach (Newell and Simon, 1972). Using this approach, one can distinguish agents in a variety of respects, for instance, by representing them as commencing their search from different initial states or by representing agents as possessing different search heuristics, as in models developed by Hong and Page (2004), Weisberg and Muldoon (2009), and Pöyhönen (2017). Another option is to represent agents as Bayesian learners with different sets of prior beliefs or with differential access to evidence in light of which they can update their beliefs, as in models of Zollman (2010) and Angere and Olsson (2017).

In addition to modeling cognitive diversity among agents, these models typically also model the communication, integration, and iteration components of IE. At a minimum, doing this requires that agents’ behavior can be informed by the performance of others, that their behavior can in turn inform others’ behavior, and so on. Within formal approaches in diversity research, there are a variety of proposals for incorporating such social learning into heuristic search approaches (Alexander et al. 2015; De Langhe 2014; Holman et al. 2018; Hong and Page 2001, 2004; Page 2007, 2017; Pöyhönen, 2017; Weisberg and Muldoon, 2009).^10^ In Hong and Page’s (2004) model, for instance, each agent is represented as a unique set of heuristics distinguishing it from other group members. The agents communicate with each other by observing the point where the set of heuristics possessed by the previous problem solver could no longer offer any improvement. Agents then integrate and iterate by using their own heuristics to seek improvements from the starting point provided by the previous agent. The collective stops when no agent can offer any further improvement to the solution reached. The communicate–integrate–iterate dynamic is also present in other models. In Weisberg and Muldoon (2009), agents can observe results obtained by adjacent neighbors on a grid, and then choose where to go next depending on those results and whether they use a “Follower” or “Maverick” heuristic. In contrast, Zollman (2010) employs the tools of network analysis for enabling Bayesian agents to engage in social learning. Within this approach, each agent is represented as a node in a network and the communicative pathways between them are modeled as connections between the nodes. Accordingly, scientists can update their priors in light of the evidence gathered by their own actions as well as the evidence arriving from agents to which they are directly connected.

In sum, several formal models designed to study epistemic effects of diversity represent the key elements of IE. Let us consider, then, whether any of these models can study potential impacts of demographic diversity upon IE, and consequently whether they may be useful for exploring DBIE explanations or the double-edged sword. To do this, a model would need to represent diversity in two types of attributes, one that could be interpreted as demographic and another as task-related. Furthermore, the communication-integration-iteration process that comprises IE should be modulated by the demographic composition of the group. The models considered here represent diversity in task-related cognitive characteristics only (e.g., search heuristics, prior probabilities, etc.) and not in any attributes that might be interpreted as demographic, such as in-groups and out-groups. Consequently, they also do not examine how IE might depend on demographic diversity.

Nevertheless, there are interesting analogies between DBIE explanations and some of the models described above. Zollman (2010) shows that a sparsely connected network is beneficial insofar as the resulting diversity of information makes scientists less likely to converge to a premature consensus on a false belief. In other words, while dense networks can converge to the truth more quickly, they are also vulnerable to converging on false but initially promising hypotheses. These results are analogous to the in-group epistemic conformity hypothesis (Fig. 2b), according to which too much reliance on testimony of others can result in downgrading cognitive resources at one’s disposal and consequently to failures of integration. For their part, Angere and Olsson (2017) suggest that epistemic advantages can ensue from scientists being more discerning about what information they share with colleagues, for instance, by not publishing unless they are very confident of their results. This resembles one aspect of mechanism 2(a), in which observed demographic diversity causes people to expect greater cognitive diversity, which in turn causes them to analyze and assess information at their disposal more carefully before communicating to the group. But these network models do not consider the effect that demographic diversity might have on network structure or expectations, although ethnic “homophily” is documented in science (Freeman and Huang 2015).^11^

In sum, while several of the models discussed here represent IE, none examine potential dependencies of IE on demographic diversity. Consequently, they do not explore the negative path of the double-edged sword and mechanisms associated with DBIE explanations. These limitations are clearly significant if these models are used to support practical or policy recommendations, as some in fact have been (cf. Page 2007, 2017). Moreover, we suggest that these limitations are also relevant for models that aim only to provide understanding of the effects of diversity on group performance. While we agree that it can be useful to drill down on a single aspect of a phenomenon in idealized isolation (cf. Singer et al. 2018, p. 5), we do not agree that this is the *only* way models can promote understanding. In some cases, exploration of complex interactions between two or more aspects of a phenomenon may be crucially important, both for practical purposes and for theoretical understanding. We claim that the relationship between demographic diversity and IE is just such a case in the context of impacts of diversity on group performance. As discussed in Sect. 2, this relationship is crucial for the double-edged sword, which is commonly used to frame empirical results in the field, as well as for the more heterodox DBIE explanations. Thus, while we recognize the value of previous models in furthering understanding of epistemic effects of diversity, we also regard inattention to the relationship between demographic diversity and IE as an important practical and theoretical limitation.

Consider how the impact of demographic diversity on IE might be modeled. One simple approach would be to introduce in-groups and out-groups to network models, where agents, for instance, are more likely to trust members of their own group. An obvious hypothesis is that mutually distrustful subgroups would have similar effects as reducing network density (cf. Zollman 2010). However, the effects may depend on a number of details, such as the relative proportions of the groups and how they are distributed throughout the network. Furthermore, distrust of out-group individuals may erode as individuals work together (van Dijk et al. 2012), and this could be modeled as well. One could also examine scenarios wherein group membership is independent of cognitive diversity (e.g., in the form of prior probabilities) as well as scenarios in which members of one group are initially inclined to believe one thing and members of the other group something else.

Of course, other modelling approaches besides network analysis could be considered. But the above brief discussion suffices to suggest that there is no inherent technical obstacle to modeling the relationship between demographic diversity and IE. Moreover, such modeling efforts could be helpful for delineating conditions in which demographic diversity is more likely to have positive versus adverse effects on group performance. As explained in Sect. 2.2, the disagreement between the double-edged sword and DBIE explanations turns on this issue. The former says that all positive effects of demographic diversity are mediated by task-related diversity, while the latter denies this and says that demographic diversity can also improve group performance by enhancing IE. Thus, models such as those sketched in previous paragraph could helpfully link philosophical and empirical diversity research.

### Diversity and objectivity

In this section, we consider Longino’s (1990, 2002) account of diversity and scientific objectivity in connection with IE. In *Science as Social Knowledge,* Longino emphasizes the social character of scientific knowledge, and offers an account of scientific inquiry as a collective practice mediated by socio-political values and interests. On this picture, scientific theories are legitimated through “critical processes involving the dynamic interplay of observational and experimental data and background assumptions” (Longino 1990, p. 13). Longino analyzes this process via a series of conditions that must be met if a scientific community is to be objective:

Scientific communities will be objective to the degree that they satisfy four criteria necessary for achieving the transformative dimension of critical discourse: (1) there must be recognized avenues for the criticism of evidence, of methods, and of assumptions and reasoning; (2) there must exist shared standards that critics can invoke; (3) the community as a whole must be responsive to such criticism; and (4) intellectual authority must be shared equally among qualified practitioners. (Longino 1990, p. 76; cf. Longino 2002, pp. 129–131)

Some additional explanation is needed to properly understand this passage.

Several pages on Longino writes that, “[T]he greater the number of different points of view included in a given community, the more likely that its scientific practice will be objective” (1990, p. 80; cf. Longino 2002, p. 131). Note that the number of distinct points of view is not one of the four criteria said to determine the extent to which a scientific community is objective. What, then, is the relationship between the number of different points of view and the four criteria? The best interpretation, we suggest, is to take the four criteria as conditions in which cognitive diversity is claimed to give rise to objective science. In other words, the proposal is that variation in knowledge, perspectives, and methods within the community stand to improve the quality of science, but only if the four criteria are satisfied. Conversely, we take it to be Longino’s position that the four criteria would be insufficient for objectivity in a community in which few distinct perspectives were present.

This interpretation suggests a link between Longino’s proposal and IE. The presence of multiple points of views indicates a context in which there are dispersed cognitive resources in the community of scientists. The background condition needed for IE to be relevant, then, is in place. Moreover, of Longino’s four criteria, two straightforwardly correspond to the communicate–integrate–iterate process of IE. Criterion 1, in which there are recognized venues for sharing criticisms, is the communication step, and criterion 3, in which the community is required to uptake and respond to criticisms, represents the integration and iteration steps. In Longino’s model, a criticism of some prevailing position or interpretation of evidence is communicated, and then other members of the community integrate this criticism with their own cognitive resources to generate a response, which is then communicated to the community, and so on. Criteria 2 and 4 are naturally interpreted as effect modifiers intended to enhance IE. More specifically, these criteria appear designed to remove two possible barriers to the integration step. The shared standards criterion precludes a situation in which a criticism is unintelligible to other community members due to a lack of shared perspectives, concepts, or methodological norms, while tempered equality of intellectual authority prohibits downgrading criticisms because of irrelevant aspects of the speaker’s social identity.

Like several of the models of diversity described in the previous section, then, Longino’s account of scientific objectivity provides a representation of IE. But like those models it is also limited with respect to considering the potential dependence of IE on demographic diversity. Specifically, Longino’s four criteria suggest an awareness of negative, but not positive, effects of diversity upon IE. Criteria 2 and 4 (shared standards and tempered equality of intellectual authority, respectively) imply that diversity can have negative effects on IE. Criterion 2 is motivated by the thought that too much cognitive diversity can inhibit comprehension of criticism, and criterion 4 by the observation that diversity of social identity or status is sometimes associated with the disregard of views expressed by outgroups. However, none of the criteria reflect the idea that demographic diversity can have positive effects on IE. In fact, the criteria significantly restrict the scope of the two DBIE explanations from Sect. 2.2.

Recall the cognitive diversity expectation mechanism represented in Fig. 2a, wherein group members take visible demographic diversity as an indicator of cognitive diversity, and conversely homogeneity to indicate its absence. This mechanism is plausibly explained by a human tendency to stereotype in which noticeable markers of social identity are treated as accurate predictors of beliefs, knowledge, perspectives, and so on (Carter and Phillips 2017). The suggestion is that such stereotyping can result in demographic diversity exerting positive effects on IE, as the expectation that socially different others hold differing views may prompt group members to assess information at their disposal more rigorously and prime them to recognize novel or dissenting perspectives. According to the in-group epistemic conformity mechanism in Fig. 2b, people are more inclined to agree with, and expect agreement from, others perceived to be similar to themselves. This can result in giving more weight than warranted to beliefs expressed by socially similar others and to a reluctance to assert dissenting information or perspectives.

The mechanisms just described conflict with Longino’s criteria of tempered equality of intellectual authority. This criterion demands that the seriousness given to a claim should depend on *what* it says, not on *who* said it, except when the identity of the speaker is an indicator of task-relevant expertise. For example, tempered equality of intellectual authority is compatible with giving an astronomer’s views about dark matter more weight than those of an anthropologist. But it is not compatible with granting more epistemic authority to male over female astronomers on this topic (cf. Longino 2002, p. 131). Yet according to the cognitive diversity expectation and in-group epistemic conformity hypotheses, individuals are likely to respond differently to claims given the social identity of the speaker, even when those identities are not linked to task-related expertise. According to cognitive diversity expectation, individuals are more likely to diligently integrate a newly communicated claim when it is made by an out-group individual—and consequently may be more likely to turn up conflicting information. According to in-group epistemic conformity, individuals are less inclined to challenge claims made by others perceived as socially similar—and consequently are morely likely to object to a claim made by someone socially different than themselves.

We suggest that two significant implications flow from the above observations. The first is that Longino’s model possesses similar limitations as the formal models discussed above. Longino’s model may be of limited use for providing practical guidance or theoretical understanding with regard to actual situations in which mechanisms identified by DBIE explanations play an important role. The second implication is that epistemic and justice-based rationales for diversity may not always go hand in hand, because some epistemically beneficial effects of diversity may depend on a less than entirely just social background. The mechanisms posited by the DBIE explanations discussed above are plausibly related to epistemic injustices. Pohlhaus argues that stereotyping is linked to epistemic injustice (Pohlhaus 2017, p. 21), and Grasswick counts violations of Longino’s ideal of intellectual authority among examples of epistemic injustice in science (Grasswick 2017, p. 317). And refraining from voicing dissenting views due to expected resistance from members of one’s identity group is an example of testimonial smothering, which “occurs because the speaker perceives one’s immediate audience as unwilling or unable to gain the appropriate uptake of proffered testimony” (Dotson 2011, p. 244).

We regard the potential divergence of epistemic and equity-based rationales for diversity as having broad philosophical interest. Epistemic injustices are typically regarded as harmful both from the perspective of ethics and epistemology. However, DBIE explanations propose that epistemically unjust social dynamics can be important for explaining how diversity has positive epistemic effects at the level of groups. That in turn suggests a new line of inter-connected research questions for philosophical work on the epistemic impacts of diversity. For example, under what circumstances are mechanisms identified by DBIE explanations epistemically unjust, and when are they likely to produce epistemic benefits? And what is the connection between social equity and the potential for diversity to enhance the epistemic performance of groups?

Philosophical inattention to such questions seems related to a general lack of awareness of DBIE explanations among philosophers. Indeed, we know of only one philosophical work that discusses DBIE explanations, namely, a recent article by Muldoon examining diversity in the context of political philosophy (Muldoon 2018). Like some proponents of DBIE explanations (cf. Phillips et al. 2009), Muldoon highlights the “pain is worth the gain” thesis of diversity effects: even when demographic diversity is linked to improved results, it is often associated with less positive subjective experiences on the part of group members. In this vein, Muldoon writes, “if we choose diversity, we are choosing a harder, more complex society that has more sources of social friction. So insofar as we believe diverse communities to be better ones, we need to find mechanisms to help guide ourselves towards an embrace of diversity without being naïve about reasons for why many would not choose it” (Muldoon 2018, p. 818). The discussion in this section suggests one source of the social frictions of which Muldoon speaks. The DBIE explanations discussed here often involve unjust social dynamics likely to pass unnoticed in socially homogeneous settings (e.g., differential responses to speakers by gender cannot be observed in an all-male group). Yet tense interactions that occur when such dynamics are brought to the fore in heterogeneous groups can also yield epistemic advantages. Social processes such as these, we suggest, merit greater attention in philosophical literature on diversity in science.

## Conclusions

In this work, we have explored connections between empirical and philosophical research on epistemic impacts of diversity. Specifically, we claim that the relationship between demographic diversity and IE, while crucial to explanations found in the empirical literature, has not been adequately examined by philosophers. We aim to have made three primary contributions in connection with this.

1. We provide a detailed philosophical account of IE that can be used to clarify explanations in the empirical literature and to bring philosophical and empirical work on diversity in closer contact with one another. We define IE as consisting of a communication-integration-iteration process against a background of dispersed cognitive resources.
2. Given this, we consider the extent to which some prominent philosophical accounts of diversity in science can represent the relationship between demographic diversity and IE. These accounts generally do not consider what we call DBIE explanations, wherein demographic diversity exerts positive effects on IE and thereby on group performance. We suggest ways that formal models could be modified to do so, and thereby to shed light on when demographic diversity improves group performance and when it has the opposite effect.
3. We explain how some DBIE explanations of positive epistemic effects of diversity depend on epistemically unjust social dynamics. This raises the possibility that, under some circumstances, epistemic injustices may have positive group-level epistemic effects, an idea that to our knowledge has not been previously discussed in the philosophical literature.

In sum, both empirical and philosophical research on epistemic effects of diversity stand to benefit from being brought into closer contact.

## Footnotes

1 It is common to distinguish between groups and teams. Both involve a collection of people working jointly on a common task, but teams have more integration and joint commitment to goals among members than groups. To avoid repeated cumbersome phrases such as “groups and/or teams,” we follow McGrath et al. (1995) in treating teams as a subset of groups.

2 Oncofertility is a field dedicated to research on fertility treatments for cancer survivors.

3 Different measures of performance are also relevant, as discussed below.

4 See Webber and Donahue (2001) for an exception.

5 However, see Byron and Post (2016) for a meta-analysis that finds a positive association between gender and performance.

6 Other challenges to the double-edged sword exist. For example, some question whether demographic diversity promotes task-related diversity (Horwitz and Horwitz 2007), and others suggest that job-related diversity can also reduce group cohesion (Mitchell and Boyle 2015).

7 Similar observations can also be found in philosophical discussions of diversity in science (cf. Fehr 2011, pp. 147–148).

8 An analogous phenomenon is found in literature on polarization: namely, group polarization is more likely when members see one another as similar (Sunstein 2002, pp. 180–181).

9 These other issues include the justifiability of idealizations, and whether the model is robust when the idealizing assumptions are relaxed (Muldoon and Weisberg 2011; Thoma 2015; Pöyhönen 2017; Grim et al. 2019).

10 Some of these agent-based, heuristic search models characterize the landscape wherein the search takes place as “epistemic landscapes” (Weisberg and Muldoon, 2009; Pöyhönen 2017), while others represent it in terms of “tunably rugged landscapes” of the sort used in NK models (Hong and Page 2004; Holman et al. 2018). However, this difference is not germane to our discussion.

11 A model developed by Rubin and O’Connor (2017) does study the effects of social majority and minority groups on collaboration networks and equity of credit, but does not link these dynamics to group performance.
